# A Fused Deep Learning Architecture for the Detection of the Relationship between the Mandibular Third Molar and the Mandibular Canal

**DOI:** 10.3390/diagnostics12082018

**Published:** 2022-08-20

**Authors:** Cansu Buyuk, Nurullah Akkaya, Belde Arsan, Gurkan Unsal, Secil Aksoy, Kaan Orhan

**Affiliations:** 1 Department of Dentomaxillofacial Radiology, Faculty of Dentistry, Istanbul Okan University, 34947 Istanbul, Turkey; 2 Department of Computer Engineering, Faculty of Engineering, Near East University, 99138 Nicosia, Cyprus; 3 Department of Dentomaxillofacial Radiology, Faculty of Dentistry, Istanbul Medeniyet University, 34720 Istanbul, Turkey; 4 Department of Dentomaxillofacial Radiology, Faculty of Dentistry, Near East University, 99138 Nicosia, Cyprus; 5 Department of Dentomaxillofacial Radiology, Faculty of Dentistry, Ankara University, 06560 Ankara, Turkey; 6 Medical Design Application, and Research Center (MEDITAM), Ankara University, 06560 Ankara, Turkey; 7 Department of Dental and Maxillofacial Radiodiagnostics, Medical University of Lublin, 20-059 Lublin, Poland

**Keywords:** deep learning, segmentation, third molar, mandibular canal, panoramic radiography

## Abstract

The study aimed to generate a fused deep learning algorithm that detects and classifies the relationship between the mandibular third molar and mandibular canal on orthopantomographs. Radiographs (*n* = 1880) were randomly selected from the hospital archive. Two dentomaxillofacial radiologists annotated the data via MATLAB and classified them into four groups according to the overlap of the root of the mandibular third molar and mandibular canal. Each radiograph was segmented using a U-Net-like architecture. The segmented images were classified by AlexNet. Accuracy, the weighted intersection over union score, the dice coefficient, specificity, sensitivity, and area under curve metrics were used to quantify the performance of the models. Also, three dental practitioners were asked to classify the same test data, their success rate was assessed using the Intraclass Correlation Coefficient. The segmentation network achieved a global accuracy of 0.99 and a weighted intersection over union score of 0.98, average dice score overall images was 0.91. The classification network achieved an accuracy of 0.80, per class sensitivity of 0.74, 0.83, 0.86, 0.67, per class specificity of 0.92, 0.95, 0.88, 0.96 and AUC score of 0.85. The most successful dental practitioner achieved a success rate of 0.79. The fused segmentation and classification networks produced encouraging results. The final model achieved almost the same classification performance as dental practitioners. Better diagnostic accuracy of the combined artificial intelligence tools may help to improve the prediction of the risk factors, especially for recognizing such anatomical variations.

## 1. Introduction

Extraction of the mandibular third molar (M3) is a common dentoalveolar surgical procedure [1]. Inferior alveolar nerve (IAN) injury is one of the undesired complications encountered during M3 extraction [2]. Postoperative IAN injury is reported ranging from 0.26% to 8.4% in the literature [3]. Despite the injury being frequently transient, it may result in uncomfortable total or partial loss of sensation [4]. The risk assessment of this multicausative condition should include anatomical, surgical, and radiological factors. The injury risk is increased by advancing age because of the completion of the apex, strengthened bone density, and weakened bone elasticity [5]. Also, the eruption status, the level of impaction, presence or absence of cortex between mandibular canal (MC) and M3, IAN exposure during surgery, bone fracture or hemorrhage intraoperatively, and maximum mouth opening are some of the other variables [3,5]. The impaction’s position and depth are part of the predictive radiographic signs. It was reported that there is a higher risk of neurosensory injury after extraction of horizontally impacted M3s compared with other types of impaction, and deeper impaction levels lead to an increased risk of nerve damage [6]. Among these risk factors, the proximity of the root of M3 to the MC is reported to be the most predictive factor for IAN injuries [3]. Therefore, it is crucial to understand the relationship between M3 and MC radiologically before surgery to minimize the risk.

Because of the allowing of the evaluation of physiological and pathological conditions in the maxillary and mandibular dentoalveolar area, the initial diagnostic imaging modality is orthopantomograms (OPG) in dentistry. The evaluations of the eruption status, the root curvature, the relationship describing the extent of IAN, and the M3 root overlap of MC are considered the main prognostic factors of IAN injuries on OPG images [3]. Considering that M3 impaction is a common condition, an automated image classifier on OPGs may contribute to easier treatment planning, thus facilitating the risk assessment, and reducing human mistakes, especially in busy dental clinics.

Deep learning is increasingly beneficial in dentistry with the ability to process big data for analyzing, diagnosing, and disease monitoring [7]. Convolutional neural networks (CNN) are one of the types of deep neural networks specialized to deal with radiographic images [8]. The potential applications of neural networks in the field of dentistry are the prediction of possible diseases or clinical conditions. In dentomaxillofacial radiology, CNNs are used for the detection, segmentation, and classification of dental structures or conditions [7].

There are many algorithms used in medical image segmentation and classification. Each one has its advantages, disadvantages, and computational ability [9,10]. The review of the literature reveals that one of the disadvantages of isolated CNN architectures is that the outcomes are not convincing for complex radiological tasks [9,10,11]. The U-Net architecture is one of the most applied CNNs in medical image segmentation and the AlexNet is one of the most efficient architectures in image classification [9]. The purpose of the present study was to generate a fused deep learning algorithm that detects and classifies the relationship between the M3 and MC automatically on OPGs to build a final model with advanced diagnostic accuracy.

## 2. Methods and Materials

This study was conducted in compliance with the 1964 Helsinki declaration on medical research ethics. The Institutional Ethics Committee (Istanbul Okan University, Istanbul, Turkey) approved this retrospective analysis and informed consent was not required because the data were anonymized (Decision no. 56665618-204.01.07).

### 2.1. Preparation of Dataset

OPGs of patients who attended the Dentomaxillofacial Radiology Department of Istanbul Okan University between 2017–2020 years were randomly selected. All OPGs were acquired with the Planmeca Promax 2D S3 unit (Planmeca, Helsinki, Finland) with the standard exposure protocol (66 kVp, 8 mA, 16 s) according to the manufacturer’s recommendations. The inclusion criteria were the presence of at least one M3 with fully formed apices. The M3s with the presence of a pathology such as chronic apical osteitis, hypercementosis, or cysts; M3s with incomplete root development; horizontally impacted M3s; and M3s having untraceable relation with the MC were excluded. The OPGs with imaging artifacts were also excluded from further analysis. In total, 1950 OPGs were evaluated and 70 of them (3.59%) were excluded. A total of 1880 OPGs that meet the inclusion criteria were selected (ages ranged from 18 to 73 years). Before the analysis, all OPGs were pseudonymized.

**Figure 1:**
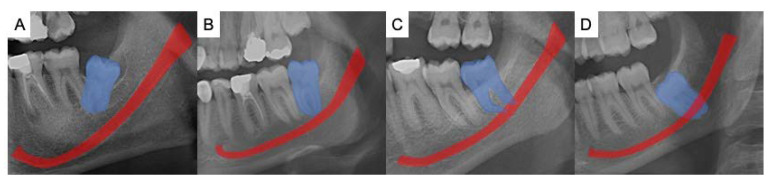
The examples for the classification of the relationship between the mandibular third molar (blue area) and mandibular canal (red area). (**A**) represents class I where there is no connection between the root tip and MC. (**B**) represents class II where the direct contact of M3 with the superior cortical boundary of the MC. (**C**) represents class III where the M3 and MC are superimposed and the (**D**) represents class IV where the root tip is under the inferior cortical line of MC.

The data set was divided in half for each observer, and two independent dentomaxillofacial radiologists annotated the MCs and M3s in the OPGs via the annotation tool MATLAB (The MathWorks, Inc., Natick, Massachusetts, USA). Under dim light conditions, both observers used different but analogous monitors for the labeling task (2560 × 1600 pixels, 13-inch high-resolution Retina display MacBook Air laptop, California, USA). Both left and right M3s and MCs were labeled. The same observers also classified each labeled region for the extent of M3 root-tip overlap with the MC according to the published method [12]. The classification was as follows; I: No connection, II: Direct contact with superior cortical boundary, III: Superimposed, IV: Root tip is under the inferior cortical line (Figure 1).

The observers re-evaluated the identical 20% of the data separately one week after the first annotation to measure the reliability. For the reproducibility of the data, each observer re-classified 20% of the randomly selected data two weeks after the first evaluation. These OPGs were selected with computer-generated random numbers. After the preparation of the dataset, the labeled OPG data were randomly split into a training group (80%), and a testing group (20%). For the classification of the relationship between the M3 and MC, the data distribution used in the AlexNet was as follows: 25.82% in class I, 29.00% in class II, 36.74% in class III, and 8.43% in class IV. The detailed data distributions of U-net and AlexNet models for training and testing sets were shown in Table 1.

### 2.2. Neural Network Architecture

**Figure 2:**
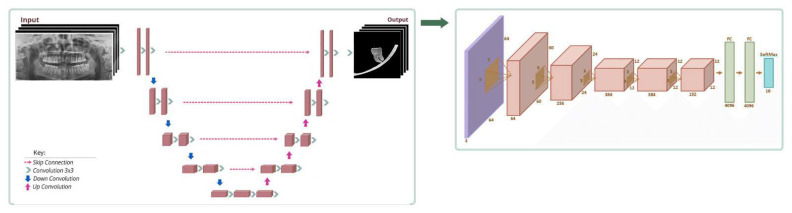
The workflow for the segmentation and the classification processes of mandibular third molar and mandibular canal. The first schema represents the encoder-decoder style network of U-Net. The output of this network feeds the AlexNet architecture which is seen in the second diagram.

The proposed architecture is summarized in Figure 2. The present algorithm applied in the study uses two separate deep neural networks by feeding the output of the first neural network into the second neural network. The first neural network is based on U-Net architecture. This network is used to locate the region of interest (ROI) M3-MC on the OPG. The output of this network is a segmentation mask containing M3 and MC on the image, a smaller image is created using this segmentation mask. This allows the algorithm to reduce the feature size before classification so that the classification network only deals with the ROI instead of the whole OPG. The second neural network is based on AlexNet architecture; its input is the ROI image and its output is the classification of inferior alveolar canal overlap.

### 2.3. Model Pipeline

The proposed approach is composed of the following steps,

Each OPG is split into 256 by 256 patches.

Each patch is segmented using a U-Net-like architecture.

Patches are combined to recreate the segmented OPG.

From the segmented OPG, the left and right molars are isolated.

A 256 by 256 patch is cropped and centered on each left and right molar.

Image augmentation is applied to further increase the number of samples for classification. Each patch is flipped on the horizontal axis.

This segmentation mask from the above step is run through a classification network based on AlexNet which results in the classification of inferior alveolar canal overlap (Figure 2).

### 2.4. U-Net

U-Net is a convolutional neural network, designed for biomedical image segmentation. It consists of an encoding and a decoding path which gives it the u-shaped architecture. The encoding path is a typical convolutional network that consists of applications of convolutions, followed by a Rectified Linear Unit (ReLU) and a max-pooling layer. During the encoding stage, the spatial information is reduced while feature information is increased. The decoding pathway combines the feature and spatial information through a sequence of up-convolutions and skips connections with high-resolution features from the encoding path.

### 2.5. AlexNet

AlexNet is a convolutional neural network that is designed for image classification. It is made up of eight layers, the first five are convolutional layers, some are followed by max-pooling layers, and the last three are fully connected layers. The output of the U-Net network is fed into AlexNet architecture to classify each image patch into 4 categories.

### 2.6. Implementation

The proposed algorithm was based on the MATLAB implementation of U-Net and AlexNet. All training and experiments were performed using NVIDIA^®^ GeForce^®^ RTX 2080 Ti GPU (NVIDIA, California, USA). For segmentation, the network was trained using SGD Adam optimizer. As a loss function cross-entropy was used. The batch size was set to 16. The learning rate was set to 10−4. For the classification, the network was trained using SGD Adam optimizer. As a loss function cross-entropy was used. The batch size was set to 32. The learning rate was set to 10−3.

Additionally, three dental practitioners (with experiences ranging from 2 to 30 years) were asked to classify the same test data to compare the classification performance of the model. After the used classification method was introduced, they performed the classification task on the same analogous monitors with the observers under dim light conditions. Their success rate was assessed using the Intraclass Correlation Coefficient (ICC) and discussed independently.

### 2.7. Statistical Analysis

Statistical analyses were conducted on SPSS version 21.0 (SPSS 21.0 Software Package Program, Inc., Chicago, IL, USA). ICC and Cohen’s kappa was used to measure the intra- and inter-reliability of the observers. For quantifying the performance of our segmentation model accuracy, intersection over union (IoU) and dice coefficient metrics are computed, for quantifying the performance of the classification model, accuracy, specificity, sensitivity, and area under curve (AUC) metrics are computed.

Accuracy is a metric that describes how the model performs across all classes. It is defined as the ratio between the number of correct predictions to the total number of predictions. IoU is a metric that describes the overlap of prediction and ground truth, in our case, this is the ratio of the network’s prediction and radiologists’ golden standard. The dice score is similar to the IoU but gives more weight to the intersection. AUC represents the degree or measure of separability. It tells how much the model is capable of distinguishing between classes.

Sensitivity (True Positive Rate) is the ability of a test to correctly identify images with their class. Specificity (True Negative Rate) is the ability of a test to correctly identify images without their class. The positive predictive value (PPV) shows that what percentage of data the model calls positive is positive. Similarly, the negative predictive value (NPV) shows the proportion of truly negative data among data that the model calls negative. Metrics were calculated by the following formulas: *Sensitivity = TP/(TP + FN); Specificity = TN/(FP + TN); PPV = TP/(TP + FP); NPV = TN/(TN + FN)*. In these formulas; classes defined by the observers were accepted as the ‘‘true’’ class. Thus, the true positive value (TP) and true negative (TN) value indicate the number of data belonging to the correctly classified positive and negative classes, respectively. The false positive (FP) value indicates the number of data that belongs to the negative class but is called positive by the model. Similarly, the false negative (FN) value is the number of data that belongs to the actual negative class that is incorrectly predicted by the model.

## 3. Results

The dataset contained 2893 image patches among 1880 OPGs. The training dataset of U-net contained 1504 images, and the testing dataset contained 376 images. ICC were 0.981 and 0.966, respectively indicating excellent reliability for each observer. The weighted Cohen’s kappa ± standard error was 0.954 ± 0.044 which represented an almost perfect agreement between observers.

Our segmentation network based on U-Net achieved an accuracy of 0.99 and a weighted IoU score of 0.98, an average dice score of 0.91 for the test set. Our classification network based on AlexNet achieved an overall accuracy of 0.80 across test images. Per class sensitivity, specificity, positive predictive value, and negative predictive value results were shown in Table 2. We achieved an AUC score of 0.85.

The test data was evaluated by three dental practitioners and the ICC were 0.951, 0.951, and 0.936, respectively indicating excellent reliability for each observer. The weighted Cohen’s kappa ± standard error was 0.864 which represented a good agreement between three observers. The most successful dental practitioner, the one with over 20 years of experience, achieved a success rate of 0.79 which can be compared with the accuracy of our algorithm.

## 4. Discussion

The current study proposed a fused deep learning architecture as a clinical decision support system for the detection and classification of the proximity of the M3 to MC. In this two-step deep learning implementation, first, the ROIs containing M3s and MCs has segmented from the large OPG images, then the relationship between those structures was classified. Besides, the test set has been re-tested by independent dental practitioners. The findings of the segmentation model (accuracy of 0.99, a weighted IoU score of 0.98, and an average dice score of 0.91) showed that the first network achieved a high detection performance. After feeding the second model with the output of the segmentation model, an accuracy of 0.80 was obtained by the classification model. The first model lowered the computational power need of the second model and made the classification task less complex by processing small image patches rather than entire OPG images. AUC score of 0.85 pointed out that the classification network has a good differentiation capacity between the classes. Additionally, the classification scores of the algorithm and dental practitioners (success rate of 0.79) showed very similar outcomes. At this point, it is a promising result in terms of clinical decision support systems that the classification model and dental practitioners achieved almost the same performance. On the other hand, the sensitivity results of the classification model, which represent the correct identification of the images according to the classes, were lower than the specificity findings. The imbalanced sample size between the classes (Class III > Class II > class I > class IV) may have caused this difference.

**Figure 3:**
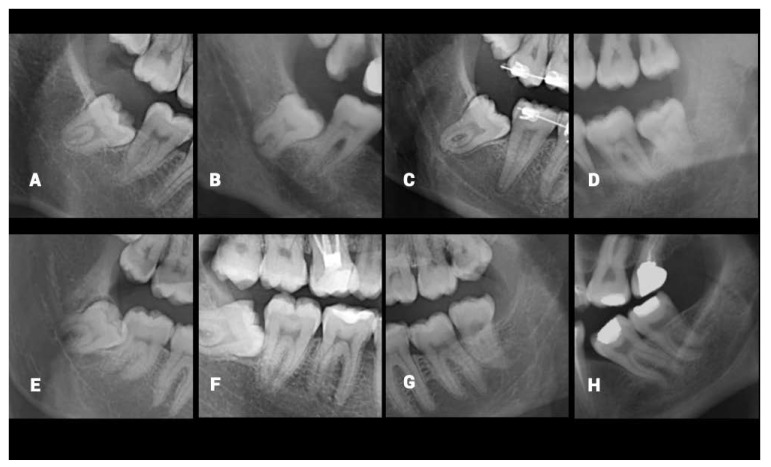
Examples of the misclassified mandibular canal and mandibular third molar overlap by the algorithm and the possible characteristics that cause misclassification: (**A**), Ill-defined superior border of MC; (**B**), Narrowing of the MC; (**C**), Dilacerated root morphology of M3; (**D**), The ghost image of the opposite inferior border of mandible; (**E**), Superimposition of the lateral border of the tongue; (**F**), Failed segmentation; (**G**), Bifid MC; (**H**), Superimposition of the mylohyoid ridge.

The dentomaxillofacial radiologists conducting the present study evaluated the misclassified cases and noted some particular radiological limitations caused by superimposition or uncertainty in the ROI. The main characteristics of those limitations were the ill-defined superior border of MC, narrowing of the MC, dilacerated root morphology of M3, the ghost image of the opposite inferior border of the mandible, superimposition of the lateral border of the tongue, failed segmentation, bifid MC variation, and superimposition of the mylohyoid ridge. The characteristics were depicted in Figure 3. The same limitations were asked of dental practitioners and they all stated that the cases in which the superior cortical border of MC is not clear, were the hardest to classify. Despite the M3s having untraceable relation with the MCs being excluded from the dataset by the specialists, it is clear that the high number of radiographic variations is a factor that makes it difficult to determine the MC-M3 relationship.

Among the M3 segmentation studies, Fukuda et al. [13] aimed to compare the diagnostic performance and consistency of AlexNet, GoogLeNet, and VGG-16 models in two different image patches with sizes of 70 x 70 pixels (AUC: 0.88–0.93) and 140 x 140 pixels (AUC: 0.75–0.90) in the evaluation of M3 and MC on OPGs. They stated that the smaller patch sizes produced better diagnostic results in all models. They also emphasized that the AlexNet was the least influenced by the difference in patch size among the three CNNs that were evaluated [13]. In the present study, the images were prepared as 256 x 256 patches for the segmentation task, and to obtain smaller data, the images were just cropped. Despite using larger image patch sizes, the similar diagnostic performance of distinguishing the classes (AUC: 0.85) was obtained through the fused U-Net and AlexNet models.

Vinayahalingam et al. [11] applied U-Net to segment the M3 and IAN on OPGs and reported 0.936 and 0.805 mean dice coefficients, respectively for M3s and IAN. Similar to our study, they stated that the lack of contrast between mandible and MC, and the shape and size variety of the MC were some of the challenges while applying the segmentation task by U-Net [11]. To improve the performance of the model and to obtain a more accurate assessment of the M3-MC relationship in the present study, larger training datasets were used to train the U-net model, and the segmented images were classified by another network based on AlexNet.

In clinical practice, OPG is a highly preferential imaging modality due to its simple accessibility, relatively easy interpretation, low cost, and less radiation dose than cone-beam computed tomography (CBCT). In the preoperative assessment of the M3s, OPGs enable the evaluation of the degree and orientation of impaction and the vertical relationship between the M3 and MC [14]. However, this imaging modality does not allow the assessment of the exact visualization of the M3s and their buccal and lingual positioning with the MC and the surrounding bone. The three-dimensional (3D) nature of CBCT imaging makes this modality very useful for the evaluation of M3s. It facilitates understanding the 3D positions of the tooth and nerve canal better by giving more detailed spatial information. Also, CBCT is accessible, less expensive, and has a lower patient dose than medical CT [14,15]. Nevertheless, the position paper prepared by the European Academy of DentoMaxilloFacial Radiology stated that CBCT should not be used routinely when evaluating M3 before extraction. The postoperative sensory disturbances of the IAN will not be decreased by CBCT imaging, and it should only be applied when there is a specific indication that cannot be answered by OPGs [16]. Taking into account the patient dose, OPG is a more beneficial modality than CBCT for the initial assessment of the M3-MC relationship. Since the OPGs are still the standard imaging modality, many dentists evaluate the relationship between M3 and MC on OPGs considering the predictive factors of IAN injury such as eruption status, the pattern of impaction, or the radiographic signs [3]. In light of all this knowledge, designing an OPG-based clinical decision support system rather than CBCT was preferred in the present deep learning study.

3D imaging is another workspace in terms of artificial intelligence for the same problem. Orhan et al. [17] designed a 3D study to evaluate the performance of an AI application in determining the impacted third molar teeth and the relationship with neighboring anatomical structures. Similar to the present study, their deep CNN algorithm was based on a U-Net-like architecture for the segmentation of the MCs on CBCT images. They reported a good agreement (kappa:0.762) between the manual and the AI examinations in the determination of the IAN to the M3s [12]. Liu et al. [18] reported a study on the automatic detection of MC-M3 on CBCT images. Similar to the present study design, firstly they segmented the MC and M3 with U-Net; then they fed the classification algorithm ResNet by the outputs of the first model. Their segmentation and classification scores were close to our results. Even though the CBCT images provide more detailed information about the anatomical relationships of the objects, 3D imaging segmentation and classification is a more complicated computer vision task than 2D studies. One of the main problems with those studies is having a lower number of data sets than the 2D studies due to the higher radiation dose or needing more computing power. Liu et al. [18] used slice-wise classification rather than 3D classification to expand the size of the training set, which means the classification task occurred at the 2D level.

There are some pros and cons of CNN models. Allowing the network to propagate contextual information to higher resolution layers by a large number of feature channels in the upsampling part is one of the important advantages of the U-net model. On the other hand, unbalanced data in the training process makes it harder for good generalization [19]. AlexNet is a relatively shallow network, it needs a larger convolutional kernel than other classification networks, and needs a greater amount of computational power, but it is capable of extracting rich features from images [9,20]. In the case of a few available samples, data augmentation is a solution to train the model with the desired invariance and robustness properties [21]. In the present study, the dataset had a variety of segmentation tasks and an imbalanced distribution in the groups for the classification task. Class I and class II have a close number of samples whereas Class III has the largest data sample. The smallest data was in class IV which shows the extension of the root was under the canal. To overcome this challenge, data augmentation techniques and combining two different CNNs were implemented. Nevertheless, class IV gave the lowest scores even though the reasonable sensitivity (0.67–0.86) and specificity (0.88–0.96) results of the classification model, which is more than sufficient for presurgical evaluation.

The retrospective nature of the study presented the unequal distribution of data based on the varying relation between M3 root apices and the MC. This limitation may be resolved by designing a prospective study with a balanced number of subgroups and a bigger data set. Also, multicenter studies with different OPG units may be designed to obtain more reliable data. The regions of interest, where the relation between M3 and MC was delineated, had superimpositions of such anatomic landmarks as hyoid bone, epiglottis, and the dorsum of the tongue. Such superimpositions, the supplementary branches, and variations of the mandibular canal in the retromolar area challenged the operator’s decision. Also, some exclusion criteria of the study such as the presence of pathology, incomplete root development, horizontal impaction, and the unclear relationship between the M3 and MC might lead to the proposed fused model giving different results from another dataset. Therefore standardization protocols on patient positioning should be applied to maintain image quality and magnification. Besides, 3D imaging may provide more precise outcomes since it did not have such superimpositions.

## 5. Conclusions

In this study, a fused deep learning algorithm was presented to detect and classify the relationship between the M3 and MC, which is one of the risk factors for IAN injuries. As a result, our two-step deep learning model achieved almost the same classification performance as dental practitioners. Thus, an encouraging image classifier was introduced for automatically analyzing the M3-MC relationship, a highly varying anatomical condition. Better diagnostic accuracy of the AI tools may help to improve the prediction of the risk factors, especially for recognizing such anatomical variations. In conclusion, we may suggest that combining different models for various dental tasks will facilitate and contribute to the enhancement of AI in dentistry. Future studies designed with postoperative clinical correlations with a hybrid perspective will increase the practical benefits of AI-based clinical decision support systems.

## Figures and Tables

**Table 1:** Distribution of the data for training and testing groups. For U-Net, the *n* represents the number of OPGs, whereas the *n* * represents the number of image patches for AlexNet.

	U-Net (*n*)	AlexNet (*n* *)			
		I	II	III	IV
**Training**	1504	598	671	850	217
**Testing**	376	149	168	213	27
**Total (percentage)**	1880 (100%)	747 (25.82%)	839 (29.00%)	1063 (36.74%)	244 (8.43%)

**Table 2:** Per class sensitivity, specificity, positive predictive value, and negative predictive value results.

	Class I	Class II	Class III	Class IV
**Sensitivity**	0.74	0.83	0.86	0.67
**Specificity**	0.92	0.95	0.88	0.96
**Positive Predictive Value**	0.79	0.88	0.80	0.68
**Negative Predictive Value**	0.90	0.94	0.93	0.96

## Data Availability

Not applicable.
